# The whole term efficacy of different treatments in paroxysmal atrial fibrillation in aging: a meta-analysis of randomized controlled trials

**DOI:** 10.18632/aging.202676

**Published:** 2021-03-10

**Authors:** Yinan Sun, Lu Wang, Xiaoyun Yang

**Affiliations:** 1 Department of Cardiology, Tongji Hospital, Tongji Medical College, Huazhong University of Science and Technology, Wuhan, Hubei, China; 2 Department of Oncology, Tongji Hospital, Tongji Medical College, Huazhong University of Science and Technology, Wuhan, Hubei, China

**Keywords:** paroxysmal atrial fibrillation, catheter ablation, antiarrhythmic drug, quality of life

## Abstract

Antiarrhythmic drug therapy (ADT) and catheter ablation (CA) are the main treatments for paroxysmal atrial fibrillation. However, the short- and long-term clinical efficacy of these treatments remains controversial. Our goal is to investigate efficacy and safety of the standardized treatment of elderly patients with paroxysmal atrial fibrillation (PAF). Eight randomized controlled trials on CA and ADT for treating PAF were included. Totally, 1336 patients were included. Studies on CA and ADT for treating PAF that were published between January 2005 and June 2020 in the Cochrane Library, PubMed and EMBASE were screened and identified. Atrial fibrillation-free rates and Short Form (SF-36) health score-related indexes were analyzed. Atrial fibrillation-free rates were similar in the CA and ADT groups [risk ratio (RR) 1.32; 95% confidence interval (CI) 0.96-1.82; P = 0.08] at 3 months. The CA group had a significantly higher atrial fibrillation-free rate at 6 months (RR 1.87; 95% CI 1.38-2.53; P < 0.001), 9 months (RR 2.38; 95% CI 1.43-3.96; P < 0.001), and 12 months (RR 2.21; 95% CI 1.28-3.84; P=0.005). However, there was no significant difference in terms of long-term efficacy at 24 months (RR 1.81; 95% CI 0.97-3.36; P = 0.06). The 12-month QOL physical and mental components (RR 2.41; 95% CI 0.89-3.93; P = 0.002) were significantly higher in CA group. The CA is more effective than ADT in the short-term prognosis. But the long-term prognosis of PAF needs to be verified via randomized controlled trials with longer follow-up durations.

## INTRODUCTION

Atrial fibrillation (AF) affected the quality of life (QOL) of 2 million patients in the United States and increased the risk of stroke and mortality [1, 2]. Antiarrhythmic drug therapy (ADT) to control heart rate and rhythm was the mainstay of paroxysmal AF (PAF) treatment. Treatment guidelines for elderly patients with PAF aim to reduce the frequency and recurrence rate; ADT was recommended as the first line treatment of PAF [3]. Amiodarone was the most effective ADT for PAF, but it is associated with a limited curative effect and can lead to some serious side effects [4, 5]. Catheter ablation (CA), used after ADT failure in clinical therapy [6–9], is a minimally invasive procedure used to treat PAF and associated with side effects such as pulmonary vein stenosis, tamponade, fistula, etc. In some special cases, CA was used as the first-line treatment and can also be used concurrently with ADT [10]. The efficacy of CA was controversial in patients with AF who had received first-line ADT and varied among individuals [1, 6].

Studies have shown that the control rate of AF recurrence at 6 to 12 months is only about 46% [5, 9–11] and the patients often discontinue therapy due to side effects [2, 7, 8]. It has been confirmed that the first-line use of CA without ADT can achieve a 60% non-recurrence rate and reduced the recurrence rate of PAF compared with ADT [2, 5, 10], but this result has not been confirmed in the investigation of the accumulated burden of PAF to patients [11]. Moreover, most clinical studies investigated the short-term curative effects and side effects of CA or ADT and rarely explored long-term efficacy and side effects [2, 8–10].

Therefore, our meta-analysis analyzed clinical studies using CA and ADT for managing PAF in terms of short- and long-term clinical efficacy and QOL to find differences between CA and ADT, with the aim of providing evidence on the standard treatment of PAF in elderly patients.

## RESULTS

### Included studies

**Figure 1:**
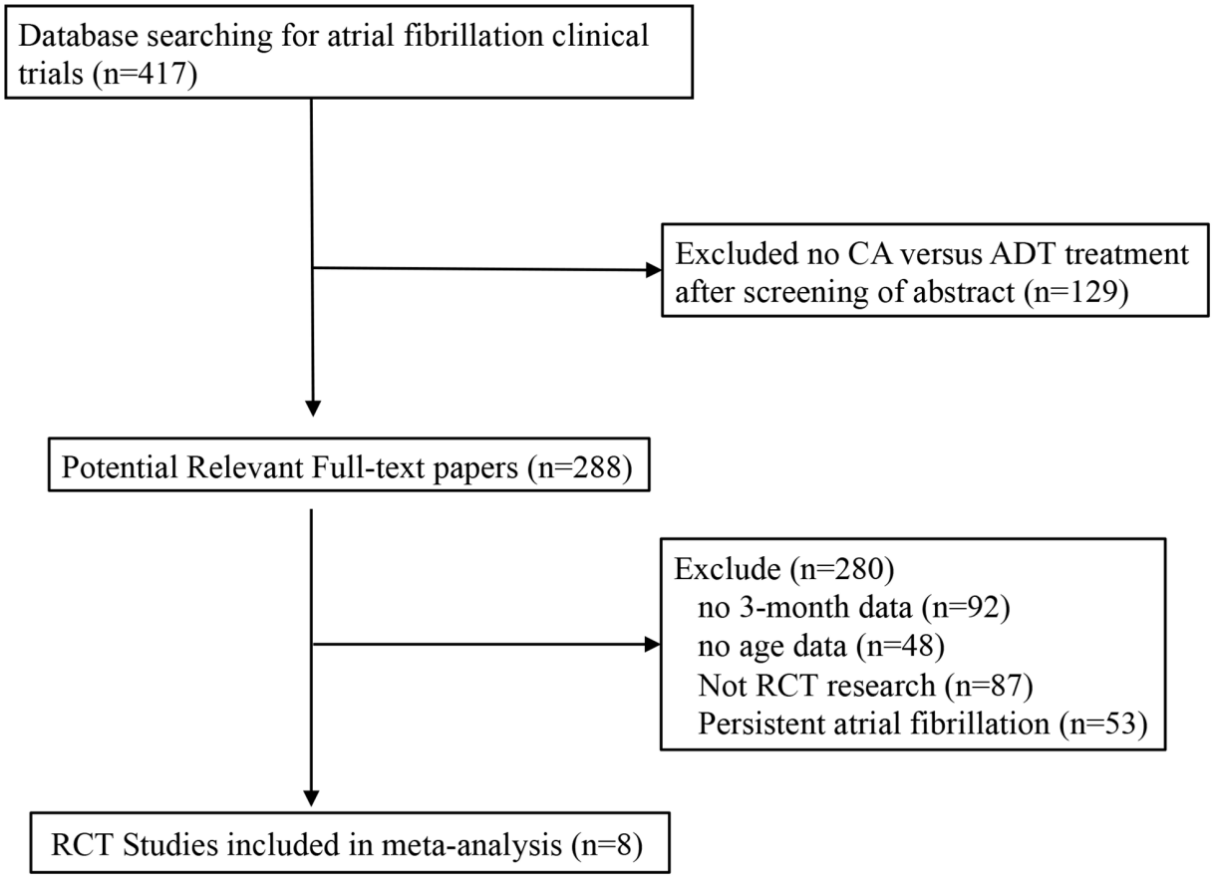
**Flow diagram of studies selection process.**

The relevant RCTs published from January 2005 to June 2020 in the Cochrane Library, MEDLINE, PubMed, and EMBASE were 288, of which 87 were not RCTs, 53 reported persistent AF, 48 had no age data and 92 had no 3-month AF-free rate data (Figure 1). A total of 8 RCT studies [2, 5, 7–12] involving 1336 patients (718 underwent CA, CA group; 618 underwent ADT; ADT group) on CA and ADT for treating PAF were included (Table 1). All studies included AF-free rate data at the 3- and 6-month follow-up; 7 had AF-free rate data at the 9-month, 5 had AF-free rate data at the 12-month, and 3 had AF-free rate data at the 24-month follow-up. For QOL, meta-analysis was conducted on the physical component summary, mental component summary, symptom frequency and symptom severity data in the groups at 3 months and 12 months.

**Table 1:** Basic characteristics of the included studies.

**Study**	**Treatment**	**Patient number**	**Follow-up**	**12-month AF free**
Carlos A. Morillo	PVI	66	24 months	73%
	ADT	61		65%
Oussama M. Wazni	PVI	33	12 months	87%
	ADT	37		37%
Pierre Jais	PVI	112	12 months	89%
	ADT	59		23%
Jens Cosedis Nielsen	PVI	146	24 months	85%
	ADT	148		71%
Carlo Pappone	PVI	99	12 months	84.8%
	ADT	99		29.3%
David J. Wilber	PVI	106	9 months	none
	ADT	61		
Evgeny Pokushalov	PVI	77	36 months	72.7%
	ADT	77		32.5%
Carina Blomstrom-Lundqvist	PVI	79	48 months	83.6%
	ADT	76		77.0%

Abbreviation: PVI, pulmonary vein isolation; ADT, antiarrhythmic drug; AF free, Atrial fibrillation-free.

### Main outcomes

### AF-free rate at 3 months

**Figure 2:**
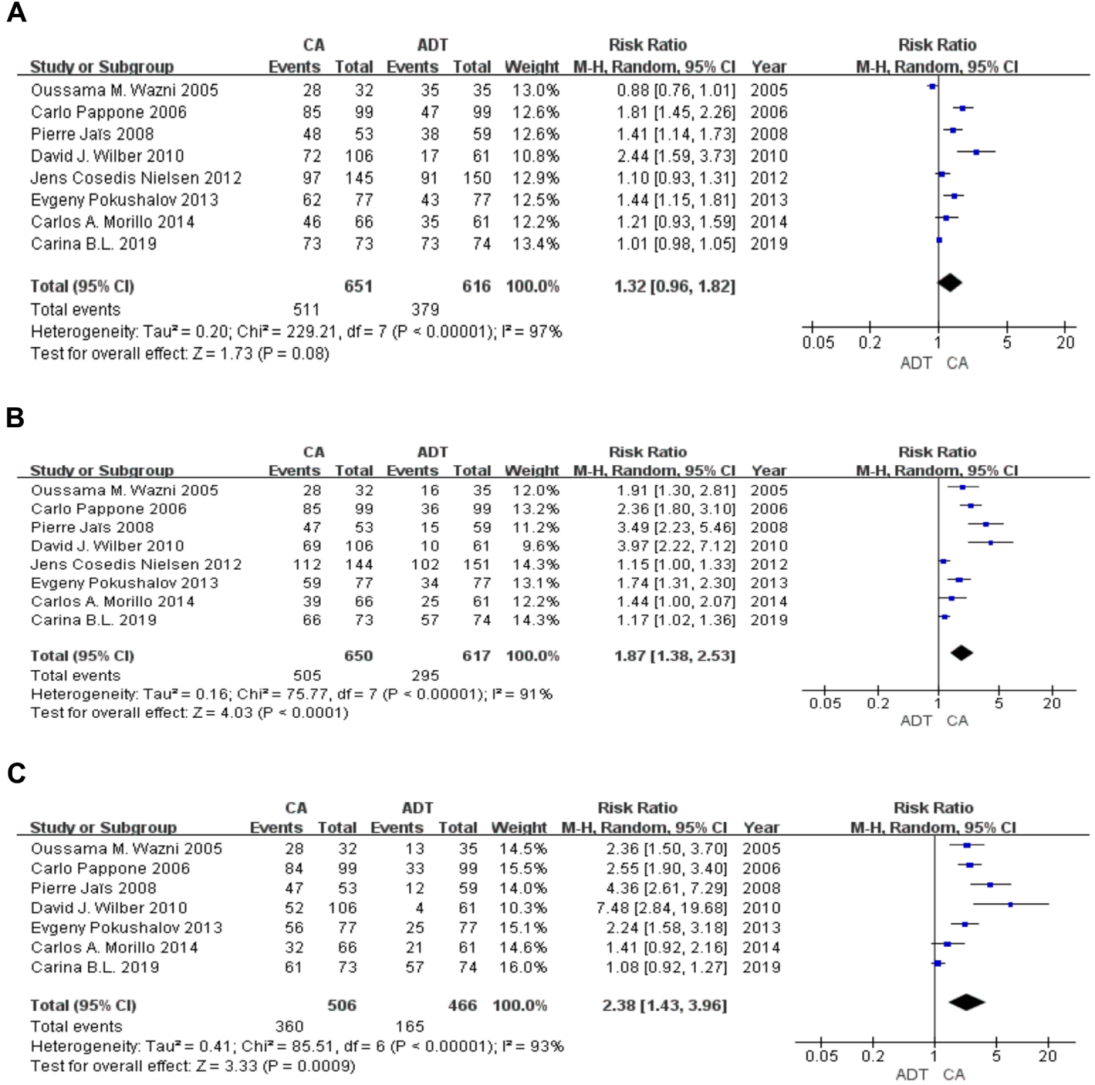
**Forest plot of the AF-free rate in the short term.** The AF-free rate was similar at 3 months (**A**) and significantly higher in the CA group than in the ADT group in 6 (**B**), 9 (**C**) months.

At the 3-month follow-up, 511 of the 651 patients in the CA group were AF-free and 379 of the 616 patients in the ADT group were AF-free. The random effects model showed a Z score of 1.73. Patients with PAF had similar outcomes in terms of AF occurrence (RR 1.32; 95% confidence interval [CI] 0.96-1.82; P = 0.08) (Figure 2).

### AF-free rate from 6 months to 9 months

At the 6-month follow-up (8 RCTs), 505 of the 650 patients in the CA group were AF-free and 295 of the 617 patients in the ADT group were AF-free. The random effects model showed a Z score of 4.03 (RR 1.87; 95% CI 1.38-2.53; P < 0.001). At the 9-month follow-up (7 RCTs), 360 of the 506 patients in the CA group were AF-free and 165 of the 466 patients in the ADT group were AF-free. The random effects model showed a Z score of 3.33 (RR 2.38; 95% CI 1.43-3.96; P < 0.001) (Figure 2).

### AF-free rate at 12months and 24 months

**Figure 3:**
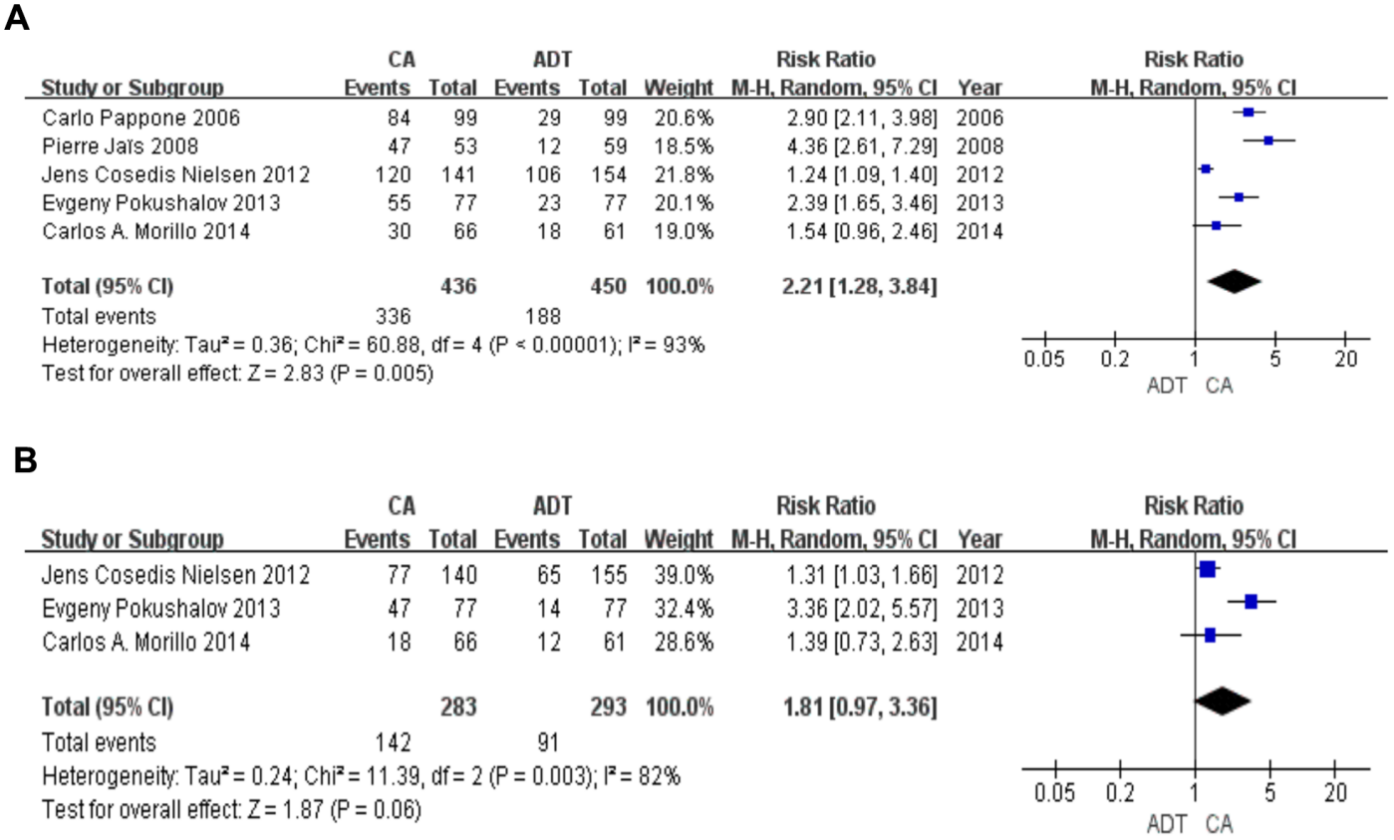
**Forest plot of the AF-free rate in the long term.** The AF-free rate was significantly higher in the CA group in 12 months (**A**). There was no statistical difference between the two groups in 24 months (**B**).

At the 12-month follow-up (5 RCTs), 336 of the 436 patients in the CA group were AF-free and 188 of the 450 patients in the ADT group were AF-free. The random effects model showed a Z score of 2.83 (I^2^=93%; RR 2.21; 95% CI 1.28-3.84; P = 0.005). These results suggest that CA resulted in a higher AF-free rate during the mid-term follow-up than ADT. At the 24-month follow-up (3 RCTs), 142 of the 283 patients in the CA group were AF-free and 91 of the 293 patients in the ADT group were AF-free. The random effects model showed a Z score of 1.87 (I^2^ = 82%; RR 1.81; 95% CI 0.97-3.36; P = 0.06). In the long-term follow-up, the CA group showed a non-significant increase in the AF-free rate compared with the ADT group (Figure 3).

### QOL

At the 3-month follow-up, QOL analysis was performed in 2 studies using the SF-36 General Health score. A total of 143 and 98 patients underwent CA and ADT, respectively, were included. In the mental component and physical component, the CA group scored significantly higher than the ADT group (RR 6.14; 95% CI 4.65-7.63; P < 0.001 and RR 5.37; 95% CI 4.01-6.73; P < 0.001, respectively). Symptom frequency scores were lower in the CA group (RR -8.7; 95% CI -14.37- -3.03; P = 0.003). There was no statistical difference in symptom severity evaluation scores between the groups (RR 8.83; 95% CI -26.84-44.50; P = 0.63). At the 12-months follow-up, QOL analysis was conducted in 2 studies using the SF-36 General Health score. A total of 202 and 205 patients underwent CA and ADT treatment. In terms of the mental component (RR 2.41; 95% CI 0.89-3.93; P = 0.002) and physical component (RR 3.32; 95% CI 1.81-4.83; P<0.001), the CA group scored higher than the ADT group.

## DISCUSSION

In this study, we found that the CA group had significantly higher AF-free rates in the early phase (6-12 months) than the ADT group. AF-free rates were also higher in the CA group than in the ADT group at the 24-month follow-up, though this difference was not significant. RCTs with longer follow-up durations of at least 2 years are recommended to verify the long-term prognosis of PAF.

In the CA group, QOL scores were higher than those in the ADT group after 3 months and 12 months of follow-up. These results suggest that in patients with PAF, primary CA can lead to better AF-free rates and QOL. Notably, it was recommended that after first-line CA failure, continuing second-line CA but not ADT resulted in higher AF-free rates. RCTs with long follow-up durations are needed to evaluate the long-term curative effect and side effects. Further studies are recommended to provide information on the predictors of the long-term prognosis of PAF. The most promising benefit reported by CA was the improvement in AF symptoms. In the early period of treatment at 3, 6, 9, and 12 months, CA reduced AF recurrence, improved the QOL, and shortened hospitalization time [1, 12]. Moreover, for some rare but important side effects, such as shock or bleeding, results were unstable, possibly due to the small sample size and short follow-up duration.

Our meta-analysis revealed that at 24 months, there was no statistically significant difference between the two treatments; therefore, evidence on the long-term benefits of CA and ADT in AF patients was limited. Most studies were not of sufficient duration (at least 2 years) to observe the long-term efficacy [2, 8–10], which is a problem that needs to be addressed in future RCTs. Moreover, the drug efficacy-cost ratio can be useful for the long-term use of ADT and short-term use of CA [13–15]. 14% of patients without recurrence of AF required second-line treatment after the 2-year follow-up, while 50% of patients relapsed in the second year after treatment with a single method [7, 13, 16]. A study [11] revealed that there was no statistically significant difference in the cumulative burden of PAF over the 2-year follow up; therefore, they recommended ADT but not CA in the early phase, which conformed to guidelines. Moreover, 36% of patients who used ADT as the first-line treatment would require second treatment with CA in the first year. A study [10] reported that ADT can lower the mortality rate and reduce side effects in the long-term. In the 12-month follow-up, 87% of patients who underwent CA were AF-free, while only 37% of those who underwent ADT were AF-free. Therefore, CA was 2.5 times more effective than ADT in controlling AF recurrence. In terms of cardiac structure remodeling, ADT had no effect compared to CA. However, death [17–19] was a risk during the entire CA procedure. Although the operational risk of CA was reduced, the reduction in mortality and stroke was rarely reported. In addition, the adverse effects of ADT, such as thyroid dysfunction, caused 23% of patients to discontinue treatment, in addition to the accumulation of more serious side effects over the long term [12, 17, 20, 21]. Although CA was superior to ADT in the first year, the long-term efficacy remains to be evaluated, which is key problem in all current RCT studies [2, 8–10].

The standard sequential therapy of CA or ADT is controversial. A study [9] included PAF patients who had failed first-line ADT. They found that CA improved symptoms, QOL, and exercise tolerance compared with ADT. They also revealed that only 23% of patients who underwent ADT showed improvement in AF symptoms even after amiodarone use during first-line ADT. The study was deficient in its small sample size, short follow-up time, and the safety of discontinuation of antiplatelet drugs in CA treatment remained to be explored. In Wilber’s research [8], CA was used after failure of first-line ADT in patients with AF symptoms. AF-free survival and control of QOL were higher after CA than after ADT. Importantly, if the response to ADT was poor in the early phase, amiodarone could only achieve 9% to 23% efficacy. The relative safety was also higher after CA, with only 6% of patients with PAF who underwent CA reporting major adverse events, including thromboembolic events, atrioesophageal fistula, cardiac perforation, phrenic nerve palsy, and death [4, 13, 22]. In the study by Pokushalov [7], after failure of first-line CA, 23% of patients who received second-line ADT progressed to persistent AF, compared with only 4% of those who receive second-line CA. After long-term observation, ADT was recommended, despite no improvement in the AF-free rate. Notably, the time of follow-up and the instruments used to evaluate AF influenced outcomes. After the 3-year follow-up, the AF-free rate in the CA group (58%) was significantly higher than that in the ADT group (12%). First-line CA was not recommended, which applied only after ADT treatment failure. Furthermore, after CA failure, secondary CA was more effective than ADT. However, partial studies included in the study did not have enough age data in detail to distinguish the elderly patients, although most of patients' age were older than 60 years old.

In conclusion, for elderly patients with PAF who underwent CA, a higher AF-free rate was obtained in the early stage. However, after 24 months, the difference in the AF-free rate was not statistically significant. Our meta-analysis revealed that after first-line CA or ADT failure, repeat CA but not ADT can result in a higher AF-free rate. RCTs are needed to evaluate the long-term curative effect and side effects. Furthermore, studies should be designed to discover new predictors for the prognosis of PAF following CA or ADT.

## MATERIALS AND METHODS

### Search strategy

This meta-analysis examined the short- and long-term efficacy and safety of CA and ADT in terms of AF-free rates and QOL scores at 3-24 months. Search terms included “paroxysmal atrial fibrillation”, “catheter ablation”, “antiarrhythmic drug treatment” and "elderly patients" to collect all relevant randomized controlled trials (RCTs) published from January 2005 to June 2020 in the Cochrane Library, PubMed, and EMBASE.

### Selection criteria and study selection

The inclusion criteria were as follows: clinical trials of PAF in which patients underwent CA and ADT, AF-free survival, and follow-up duration of more than 3 months, elderly patients (≥ 65 years old). The exclusion criteria were persistent AF, non-RCTs, no CA and ADT, no age data and no AF-free survival data. Data extraction was performed by two reviewers who independently checked for the quality and accuracy of the data. This involved identifying the disease as PAF, the CA and ADT groups, and the type of study; assessing study quality and clinical research data, the first recurrence of atrial tachyarrhythmia-free rates in 3, 6, 9, 12, and 24 months; In case of unclear or inconsistent factors, assessment and analysis were done by a third reviewer.

### Data extraction and quality assessment

Using the AF-free survival data curve of 3 to 24 months, the AF-free survival rate was extracted at 3, 6, 9, 12 and 24 months. The physical component summary, mental component summary, symptom frequency, and symptom severity data in the Short Form (SF-36) General Health score were also extracted. The research used the PRISMA Checklist and Cochrane Reviewers’ Handbook to help improve reporting quality.

### Statistical analysis

Review Manager software (version 5.2; Cochrane Collaboration, Oxford, UK) was used for meta-analysis. Heterogeneity was assessed by Cochrane χ2 statistic and I^2^ statistic. Low (I^2^ ≤ 25%), moderate (I^2^ > 25% and < 75%), or high (I^2^ ≥ 75%) heterogeneity was selected by a random effects model or fixed effects model. Efficacy results are presented in terms of risk ratio for AF-free survival rate and QOL score. All studies were assessed for publication bias using a funnel plot and Egger's test [23]. Two-tailed P values < 0.05 were considered statistically significant.
